# From Immunity to Oncology: Itaconic Acid as a Driver in HBV‐Induced HCC

**DOI:** 10.1111/jcmm.70612

**Published:** 2025-06-27

**Authors:** Shahab Mahmoudvand, Sheida Behzadi Sheikhrobat, Somayeh Shokri, Hossein Bannazadeh Baghi

**Affiliations:** ^1^ Infectious Disease Research Center Hamadan University of Medical Sciences Hamadan Iran; ^2^ Department of Virology School of Medicine, Hamadan University of Medical Sciences Hamadan Iran; ^3^ Research Center for Molecular Medicine Hamadan University of Medical Sciences Hamadan Iran; ^4^ Department of Virology School of Medicine, Ahvaz Jundishapur University of Medical Sciences Ahvaz Iran; ^5^ Department of Virology Faculty of Medicine, Tabriz University of Medical Sciences Tabriz Iran; ^6^ Immunology Research Center Tabriz University of Medical Sciences Tabriz Iran; ^7^ Infectious and Tropical Diseases Research Center Tabriz University of Medical Sciences Tabriz Iran

**Keywords:** hepatitis B virus, hepatocellular carcinoma, Itaconic acid

Dear Editor,

Itaconic acid (ITA) is an immunomodulatory mammalian metabolite secreted from primary macrophages that dramatically increases upon activation. The metabolite plays a significant role in epigenetic regulation, influencing immune responses and disease progression. In recent years, ITA has gained attention due to its anti‐microbial and immunomodulatory activities [1]. However, the ‘yin and yang’ role of itaconate should not be overlooked because it can promote tumour growth [2]. This letter offers an overview of ITA's epigenetic role, which may provide new strategies for treating hepatocellular carcinoma (HCC), particularly in patients infected with the hepatitis B virus (HBV). The interplay between HBV and host epigenetic mechanisms is crucial in developing HCC, suggesting that interventions targeting these pathways could be beneficial.

ITA has been identified as a modulator of histone modifications, particularly through a process known as lysine itaconylation. This modification plays a significant role in various biological processes, including immune responses and cancer progression [3]. Itaconate promotes the expression of immune checkpoint proteins like programmed cell death protein 1 (PD‐1) and T cell immunoglobulin and mucin domain 3 (TIM‐3) by enhancing histone modifications at the Eomesodermin (EOMES) promoter, contributing to CD8^+^ T‐cell exhaustion in HCC [4]. Research indicates that itaconate promotes CD8^+^ T‐cell exhaustion via epigenetic induction, which may exacerbate HCC development in HBV‐infected individuals. Thimme et al. [5] recently explained that CD8^+^ T cells are crucial in controlling HBV infection but are functionally impaired during chronic HBV infection.

CD8+ T cells play a crucial role in the context of HBV infection. Specifically, during acute‐resolving HBV infection, these cells serve as the primary effector cells that facilitate viral clearance and contribute to the pathogenesis of the disease. CD8+ T cell exhaustion refers to impaired function and diminished numbers of T cells, significantly contributing to advancing HBV infection [6]. Recent progress in exploring exhausted T cells during chronic HBV infection has provided novel insight into the possibility of immunotherapy for this disease [7]. Gu et al. demonstrated an epigenetic connection between itaconate and HCC, indicating that focusing on immune‐responsive gene 1 (IRG1), which is responsible for the synthesis of itaconate, or on itaconate itself could represent a promising approach for the treatment of HCC. Furthermore, combining T‐cell and anti‐PD1 therapy may offer potential curative effects [4]. Conversely, while lysine itaconylation presents a promising area of research, the complexity of viral interactions with host modifications indicates that further studies are necessary to elucidate its specific role in HBV infection and pathogenesis. Addressing this itaconate through targeted therapies may enhance treatment efficacy in HBV patients. Therefore, more research is required to elucidate this connection entirely.

## Author Contributions

**Shahab Mahmoudvand:** writing – original draft (lead). **Sheida Behzadi Sheikhrobat:** resources (lead). **Somayeh Shokri:** supervision (lead). **Hossein Bannazadeh Baghi:** writing – review and editing (lead).

## Conflicts of Interest

The authors declare no conflicts of interest.

## Data Availability

The data that support the findings of this study are available on request from the corresponding author.
